# Head Stability and Head-Trunk Coordination in Horseback Riders: The Contribution of Visual Information According to Expertise

**DOI:** 10.3389/fnhum.2017.00011

**Published:** 2017-01-30

**Authors:** Agnès Olivier, Elise Faugloire, Laure Lejeune, Sophie Biau, Brice Isableu

**Affiliations:** ^1^CIAMS, Univ Paris-Sud, Université Paris-SaclayOrsay, France; ^2^CIAMS, Université d'OrléansOrléans, France; ^3^Normandie Univ, UNICAEN, CESAMSCaen, France; ^4^ENE, Institut Français du Cheval et de l'EquitationSaumur, France; ^5^Aix Marseille Univ, PSYCLEAix-en-Provence, France

**Keywords:** head stability, postural stability, head-trunk coordination, visual information, field dependence-independence, horseback rider, expertise, riding simulator

## Abstract

Maintaining equilibrium while riding a horse is a challenging task that involves complex sensorimotor processes. We evaluated the relative contribution of visual information (static or dynamic) to horseback riders' postural stability (measured from the variability of segment position in space) and the coordination modes they adopted to regulate balance according to their level of expertise. Riders' perceptual typologies and their possible relation to postural stability were also assessed. Our main assumption was that the contribution of visual information to postural control would be reduced among expert riders in favor of vestibular and somesthetic reliance. Twelve Professional riders and 13 Club riders rode an equestrian simulator at a gallop under four visual conditions: (1) with the projection of a simulated scene reproducing what a rider sees in the real context of a ride in an outdoor arena, (2) under stroboscopic illumination, preventing access to dynamic visual cues, (3) in normal lighting but without the projected scene (i.e., without the visual consequences of displacement) and (4) with no visual cues. The variability of the position of the head, upper trunk and lower trunk was measured along the anteroposterior (AP), mediolateral (ML), and vertical (V) axes. We computed discrete relative phase to assess the coordination between pairs of segments in the anteroposterior axis. *Visual field dependence-independence* was evaluated using the Rod and Frame Test (RFT). The results showed that the Professional riders exhibited greater overall postural stability than the Club riders, revealed mainly in the AP axis. In particular, head variability was lower in the Professional riders than in the Club riders in visually altered conditions, suggesting a greater ability to use vestibular and somesthetic information according to task constraints with expertise. In accordance with this result, RFT perceptual scores revealed that the Professional riders were less dependent on the visual field than were the Club riders. Finally, the Professional riders exhibited specific coordination modes that, unlike the Club riders, departed from pure in-phase and anti-phase patterns and depended on visual conditions. The present findings provide evidence of major differences in the sensorimotor processes contributing to postural control with expertise in horseback riding.

## Introduction

Horseback riding is a challenging task that requires regulating postural balance while sitting on a moving base of support. To control their balance, riders need to adapt their movements to those of the horse while picking up information in the environment to direct the horse toward the intended goal. Stabilizing the head in this context is very challenging and yet crucial for motor performance. Because the head contains the visual and vestibular systems that play a decisive role in balance control, its stabilization in space is important for optimal processing of visual and vestibular information (e.g., Gresty and Bronstein, 1992; Amblard et al., 2001) and therefore, to provide a stable base for action (e.g., Ripoll et al., 1986; Clément et al., 1988; Pozzo et al., 1990). In the present study, we sought to assess whether (i) postural stability^1^, and more specifically head stability, is a signature of expertise in horseback riders, (ii) the contribution of visual information to riders' postural stability is reduced among expert riders in favor of vestibular and somesthetic reliance, and (iii) expert riders adopt specific postural coordination modes to preserve head stability.

Balance control involves the visual, vestibular and somesthetic systems. The contribution of vision to balance has received the greatest attention in the literature and has been tested in numerous conditions including the suppression of visual afferences by eye closure (e.g., Perrin et al., 1998; Perrot et al., 1998; Callier et al., 2001; Rougier et al., 2003), the stimulation of the central or peripheral visual field (e.g., Berencsi et al., 2005), the deterioration of visual acuity or the reduction of the visual field (e.g., Laurent et al., 1989; Schmid et al., 2008), the inclination or displacement of the visual environment (Isableu et al., 1997, 2010, 2011; Gautier et al., 2008), the selective suppression of dynamic visual cues by stroboscopic illumination (e.g., Amblard et al., 1985) or their gain in a ground optical flow (e.g., Baumberger et al., 2004). The results of these studies highlighted the importance of vision in balance control, but these conclusions should be moderated in the context of sporting expertise. Indeed, sports activities involve complex sensorimotor skills and constrain the subjects to act and process multiple information sources (proprioceptive, tactile, auditory, etc.) with a particularly high level of accuracy and rapidity.

To be efficient, the expert develops, through years of training, optimal responses to both external and internal constraints (Ericsson et al., 1993; Ericsson and Lehmann, 1996). In particular, the contribution of sensory information to postural control evolves with training and differs according to the level of practice (Era et al., 1996; Perrot et al., 1998; Bringoux et al., 2000), the type of physical activity (Hosseinimehr et al., 2009), and the specificity of gesture, support, task, or position in the environment within the same sport or sport family (Robert et al., 2004; Bizid and Paillard, 2006; Stambolieva et al., 2011). Overall, these studies showed that the contribution of vision to the regulation of postural balance tends to decrease with expertise, while somesthetic and vestibular information become more critical. For example, experts in soccer, surfing, dance, and gymnastics can use the remaining sensory modalities to compensate for a lack of vision in unstable postures (e.g., Perrin et al., 1998; Vuillerme et al., 2001a,b; Paillard et al., 2006, 2011).

Studies on horseback riding have investigated various topics such as equine gait (e.g., Galloux et al., 1994; Peham et al., 2004), horse-rider interactions (e.g., Lagarde et al., 2005; Byström et al., 2009; Wolframm et al., 2013; Münz et al., 2014), rider muscle activity (e.g., Terada, 2000; Terada et al., 2004), rider joint position (e.g., Kang et al., 2010), and rider body movements (e.g., Münz et al., 2013; Byström et al., 2015; Eckardt and Witte, 2016; Engell et al., 2016). However, very little research has been devoted to the use of sensory information in horseback riding and none has been devoted to the contribution of sensory information to rider postural stability. Some authors have suggested that expert riders use mainly proprioceptive information rather than visual information to control the horse's pace (Laurent and Pailhous, 1982; Laurent et al., 1989). Others have emphasized the importance of haptic information for coordination between the rider's movements and those of the horse (e.g., Lagarde et al., 2005). Indeed, various contacts (e.g., with the saddle, rein, stirrup) and pressures (between the rider's pelvis and the horse's saddle, primarily) are produced during the horse/rider interaction in riding. They provide rich and patterned somesthetic information (proprioceptive and tactile) that are of utmost importance to the rider in regulating and coordinating his/her movements with those of the horse. Thus, an interesting question is whether the contribution of somesthetic information to postural stability increases with expertise in horseback riding at the expense of vision, as was observed in other sports activities.

A related question concerns interindividual differences in the use of sensory information for spatial orientation, and more specifically the visual field dependence-independence (e.g., Witkin, 1950; Oltman, 1968; Paillard, 1971; Isableu et al., 1997, 2010). It has been proposed that Field Dependence (FD) or Independence (FI) reflects the weight each individual assigns to visual or non-visual information (Isableu et al., 1997, 2003, 2010, 2011; Bringoux et al., 2016). At one extreme, field-dependent subjects are affected by the surrounding visual field and are thus assumed to rely predominantly on visual information, while, at the opposite end of the continuum, field-independent subjects are less affected by the visual surroundings and so are assumed to rely more on somesthetic and vestibular cues. The influence of this perceptual typology has been observed regularly in both perceptual orientation and postural tasks (e.g., Witkin, 1950; Crémieux and Mesure, 1994; Collins and De Luca, 1995; Luyat et al., 1997; Golomer et al., 1999; Kluzik et al., 2005; Rousseu and Crémieux, 2005; Slaboda et al., 2011).

Visual field dependence-independence is of particular interest for the present study as it has been shown both to be related to expertise in sport (e.g., Liu, 2003; Guillot and Collet, 2004; Rousseu and Crémieux, 2005) and to induce interindividual differences in postural control (e.g., Golomer et al., 1999; Isableu et al., 2003, 2010). Several studies have shown that experts tend to be more field-independent in a number of physical activities such as acrobatic sports (e.g., Liu, 2003; Guillot and Collet, 2004; Rousseu and Crémieux, 2005). Moreover, some studies have highlighted a relationship between perceptual typologies and postural performance (e.g., Isableu et al., 1997; Golomer et al., 1999; Isableu et al., 2003, 2010) showing that field-dependent subjects were less stable than field-independent subjects in postural tasks, in particular when visual conditions were altered (through the inclination of the visual frame, the suppression of dynamic visual information using stroboscopic illumination or the suppression of visual information).

To date, no study has investigated interindividual differences or the relationship between perceptual typologies and sensorimotor performance in horseback riders (Olivier et al., 2012). Addressing these questions could help understand the differences between expert and non-expert riders in the weight they assign to visual information and in their ability to use non-visual information to regulate balance. Based on the results of previous studies, it can be expected that expert riders would be less dependent on the visual field, leading them to better stabilize their head compared to novice riders.

Beyond perceptual aspects, addressing the question of postural stability in riders also raises the question of the coordination modes used to regulate balance. Postural coordination during upright stance has been studied intensively in various contexts and according to different theoretical approaches (e.g., Nashner and McCollum, 1985; Assaiante and Amblard, 1993, 1995; Bardy et al., 1999; Faugloire et al., 2005). Overall, these studies have shown that head stability—and more generally postural stability—can be achieved through different coordination modes which were found to evolve with development (Assaiante and Amblard, 1995), motor learning (e.g., Zanone and Kelso, 1992; Vereijken et al., 1997; Faugloire et al., 2006, 2009), and expertise in sports activities (Marin et al., 1999; Gautier et al., 2009). In particular, Marin et al. (1999) compared the postural coordination modes adopted by novices and experts in gymnastics in terms of hip-ankle relative phase. Their results showed that increasing the difficulty of the postural task produced a change from an in-phase pattern between the ankle and the hips (almost synchronized flexion-extension of the joints) to an anti-phase pattern (joints moving in opposite directions) occurring earlier in non-gymnasts than in gymnasts. The fact that expert gymnasts were able to maintain the in-phase pattern at greater task difficulties than non-gymnasts demonstrates that expertise in gymnastics leads to a functional modification of existing postural coordination modes.

In horseback riding, riders have to anticipate and compensate for the horse's movements in a sitting posture. While the maintenance of stance in an upright posture, either on a stable or an unstable base of support, involves mainly the ankle, hip and knee joints (e.g., Nashner and McCollum, 1985; Bardy et al., 1999), riders primarily regulate balance through movements of the pelvis, trunk and neck (Vitte et al., 1995; Silva e Borges et al., 2011; Janura et al., 2015). Thus, the results obtained in studies on postural coordination in an upright stance do not apply to horseback riding situations. Interesting insights are provided by studies on postural regulation in a sitting position (e.g., Forssberg and Hirschfeld, 1994; Vibert et al., 2001; Keshner, 2003, 2004). In these studies, participants sat on a sled that was translated in anteroposterior directions (Forssberg and Hirschfeld, 1994; Vibert et al., 2001; Keshner, 2003, 2004), sideways (Vibert et al., 2001), or rotated in the sagittal plane (Forssberg and Hirschfeld, 1994). In most studies, the participants' legs and shins were resting horizontally in front of them (Forssberg and Hirschfeld, 1994; Keshner, 2003, 2004) and visual information was suppressed (Vibert et al., 2001; Keshner, 2003, 2004). Overall, the results showed that the head lagged behind the trunk in response to the perturbation (e.g., Forssberg and Hirschfeld, 1994; Vibert et al., 2001; Keshner, 2003, 2004) and that somatosensory information generated at the pelvis level, and not vestibular information from the head, appears to trigger postural responses during sitting (e.g., Forssberg and Hirschfeld, 1994; Keshner, 2003, 2004). These interesting results do not help to understand the contribution of vision because the availability of visual information was not manipulated: vision was either suppressed by eye closure (Vibert et al., 2001) or darkness (Keshner, 2003, 2004), or was available in all conditions (Forssberg and Hirschfeld, 1994). Also, the important differences between these studies and horseback riding situations in terms of the sitting position (closed vs. open coxo-femoral angle), the nature of the movements of the base of support (linear translation in the horizontal plane vs. pitch and vertical movements) and their rhythmicity (discrete vs. cyclic) do not allow to generalize these results to riders' postural coordination modes.

The purpose of the present study was to evaluate the relative contribution of visual information to head and trunk stability in Club and Professional horseback riders and the coordination modes adopted to regulate balance depending on expertise. With this aim, the participants were asked to ride a riding simulator while facing a dynamic virtual scene under four visual conditions: in normal lighting allowing the participants to access dynamic visual cues (*continuous simulated scene condition*), under stroboscopic illumination, preventing access to dynamic visual cues (*stroboscopic simulated scene condition*), in normal lighting with full visual access to the fixed surroundings but without the projected scene and thus without the visual consequences of the displacements corresponding to the context of a ride (*no simulated scene condition*) and with no visual cues (*no vision condition*).

- Our first hypothesis was that expert riders produce lower postural displacements and deploy more efficient postural control from the top of the head to the lower trunk leading them to better stabilize their head.
- Our second hypothesis was that the contribution of visual information to riders' postural stability is reduced among expert riders in favor of vestibular and somesthetic reliance, leading experts to maintain head stability better in visually altered conditions than less experienced riders. This differential reliance is also expected to be revealed by specific perceptual typologies according to expertise, with Professional riders being less dependent on the visual field than Club riders.
- Our third hypothesis was that expert horseback riders exhibit specific coordination modes to maintain a high level of postural stability, as has been observed in studies on postural coordination and expertise in other sports (Marin et al., 1999; Gautier et al., 2009).

## Methods

### Participants

Twenty-five participants were divided into two groups based on their level of horseback riding expertise. The characteristics of the two groups of participants are presented in Table 1. One group was composed of 12 elite Professional riders who specialized in show jumping and cross country riding. These members of the French National Horseback Riding School had a minimum of 20 years of practice and had participated in international competitions. The other group was composed of 13 Club riders who were ranked “Galop 5” by the French Riding Federation and had no particular specialty in any of the equestrian sports. Some of them had participated in competitions at a regional level. All of the participants were novices in the use of a riding simulator.

**Table 1:** **Mean characteristics of the Professional and Club riders (standard deviation in parentheses)**.

	**Professional riders**	**Club riders**
Number of participants	12 [2 women]	13 [7 women]
Age	38.33 years (7.05)	29.85 years (6.07)
Height	179.58 cm (8.47)	171.23 cm (10.43)
Weight	70.75 kg (8.62)	64.54 kg (9.65)
Years of practice	29.67 years (5.48)	10.23 years (6.02)
Years of practice in competition	16.67 years (6.14)	1.54 years (2.18)
Amount of practice per week	36.17 h/week (6.56)	1.31 h/week (1.75)

All of the participants had normal or corrected-to-normal vision, and reported no balance disorder, injury or pathology that might affect their ability to perform tests on a riding simulator. Local ethical approval from the Université Paris-Sud EA 4532 ethics committee was granted for this study. Each participant signed an informed consent statement after receiving oral and written descriptions of the procedure.

### Apparatus

**Figure 1:**
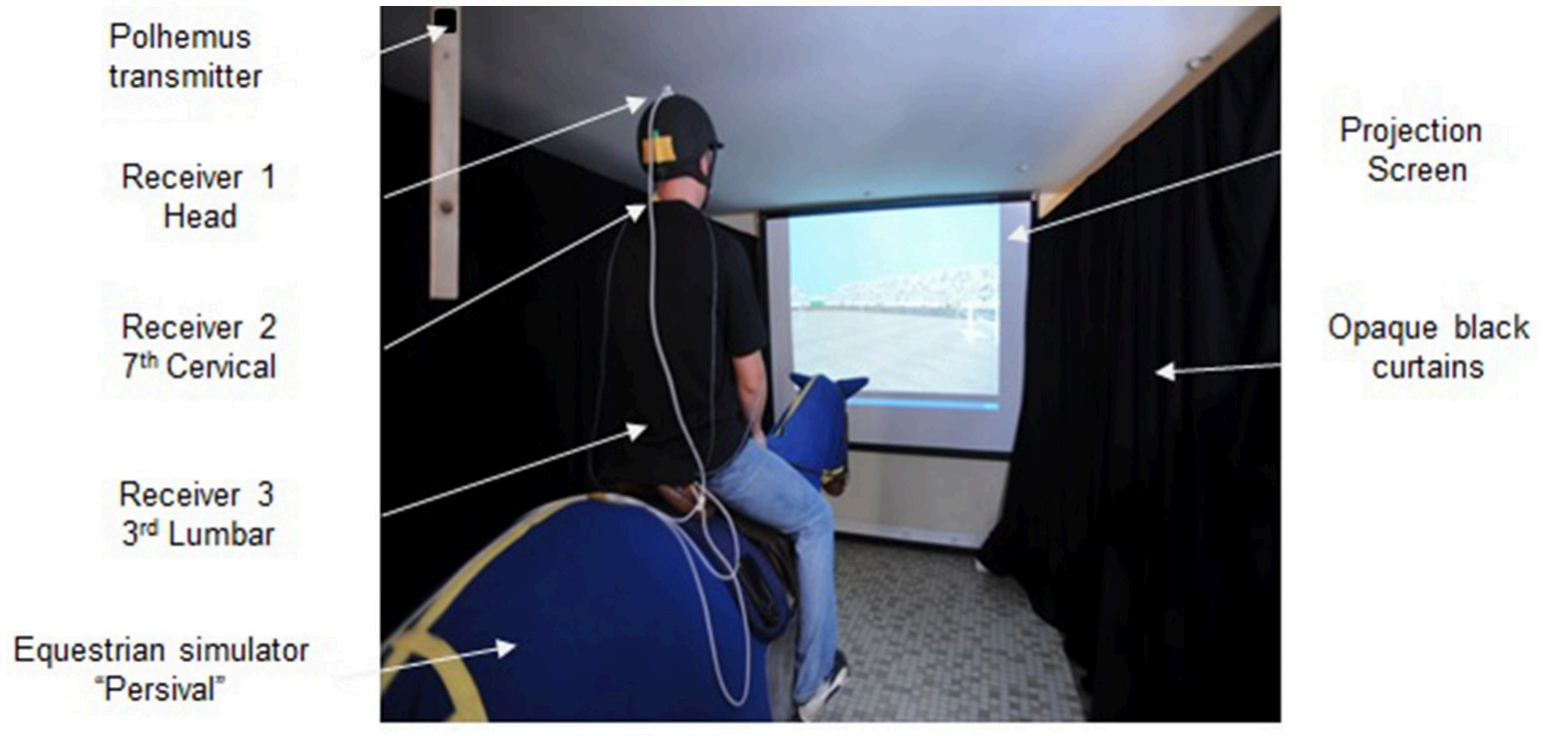
**Experimental set-up**.

Figure 1 illustrates the set-up used in this experiment. The participants rode the riding simulator Persival (Persival Industrie, Saumur, France) from the French National Horseback Riding School at a simulated gallop (stride cycle frequency of 1.4 Hz, vertical displacement amplitude of 17 cm). The use of a riding simulator ensured that the same motion of the base of support was applied to every participant. Displacements of the participants' head and trunk were measured with an electromagnetic tracking system (Fastrack, Polhemus Inc., Colchester, VT, USA), sampled at 40 Hz. Three receivers were placed on the participants: on the top of the head, on the seventh cervical vertebra (C7), corresponding to the base of the neck, and on the third lumbar vertebra (L3), which corresponds to the center of the lordotic curve of the lower back. The receiver on the top of the head was attached to the rider's helmet and the other two were attached directly to the skin using double-sided adhesive and medical cloth tape. The transmitter was located 90 cm above and 35 cm behind the back of the simulator saddle, on a shelf attached to the ceiling. The receivers attached to the head, C7, and L3 were within 52, 72, and 112 cm of the transmitter, respectively, leading to a positional resolution of 0.0025, 0.0163, and 0.095 cm, respectively (the resolution of electromagnetic tracking system measurement is affected by the distance between the transmitter and the receiver).

The participants sat on the saddle of the Persival simulator at a distance of 3.20 m from the projection screen (1.92 × 1.36 m), creating a visual angle of about 33.40° horizontally and 23.99° vertically. In order to minimize peripheral visual information, opaque black curtains were placed parallel to the rider's line of vision on either side of the experimental set-up from the edges of the projection screen to the back of the simulator. SimPiste software (Persival Industrie, Saumur, France) was used to create a computer-generated movie projected on the screen. The resulting 3D animated scene represented a classic situation of a ride in a show jumping arena from the rider's viewpoint and was synchronized with the simulator's movements. The visual environment included several fences around which the horse-rider pair moved at a gallop. Thus, the visual scene simulated displacement around the fences and the mechanical movements of the simulator maintained a gallop pace with no jumps.

### Procedure and experimental design

The experimental session began with the assessment of the participants' visual field dependence using a portable Rod and Frame Test (RFT; Oltman, 1968). In this test, participants are required to adjust a rod enclosed within a square frame on the physical vertical. The frame and the rod were tilted 18° clockwise or counterclockwise from the vertical, where the frame effect has been found to be maximal (Zoccolotti et al., 1993). Each of the four resulting conditions was presented five times, resulting in 20 randomized trials. Clear and stable differences have been found among subjects' scores on the RFT and have led to the establishment of the well-known dimension of “field dependence–independence” (Oltman, 1968; Gueguen et al., 2012): Field Dependent participants (FD) align the rod on the framework, whereas Field Independent participants (FI) align the rod on the gravitational vertical.

Next, the participants were invited to mount the riding simulator and were equipped with the Fastrack receivers. After a short period of familiarization (30 s) with the simulator, four visual conditions were presented in a randomized order as separate 50-s trials. The mechanical horse's movements were similar in every visual condition and reproduced a gallop gait. The participants were instructed to “look straight ahead and to stabilize their posture as in real practice” for each of these conditions.

– In the *no simulated scene condition* (No scene), the virtual scene was not projected and the participants faced the white projection screen under normal lighting. In this condition, continuous visual information was available from the fixed surroundings, constituting a stable reference frame for postural control.
– In the *continuous simulated scene condition* (Cont scene), the animated virtual scene from SimPiste was projected on the screen with normal lighting. In this condition, the visual scene reproduced what a rider sees in the real context of a ride in a show jumping arena, with forward displacements and turns in the virtual environment.
– In the s*troboscopic simulated scene condition* (Strob scene), the animated virtual scene was projected on the screen but its dynamic visual cues were eliminated by stroboscopic illumination (2.8 flash/s). In this condition, visual information was reduced to static visual cues in order to evaluate the various contributions of static visual cues vs. dynamic visual cues, available in normal lighting, to postural stability (Amblard and Crémieux, 1976; Amblard et al., 1985).
– In the *No vision condition* (No vision), the participants wore opaque glasses (swimming goggles covered with opaque adhesive tape) that prevented access to environmental information. This condition assessed the general contribution of vision to the riders' postural stability.

The Strob scene and No vision conditions corresponded to visually altered conditions in which the availability of dynamic visual cues (Strob scene) or the totality of the visual scene (No vision) was suppressed. The No scene condition was used as a reference condition in which postural control was expected to be facilitated by the presence of fixed surroundings (e.g., Lee and Lishman, 1975; Guerraz et al., 2001).

The participants were given a few minutes' break between conditions. The experiment took about 40 min to complete from the RFT to the last vision condition on the simulator.

### Data analysis

The raw position data collected by the magnetic tracking system were processed and analyzed using Matlab software (R2009b, The MathWorks Inc., Natick, MA, USA). In order to eliminate the initiation of the simulator's motion and participants' adaptation phase to it, we considered position data from the fifth second to the end of each trial. Two types of dependent variables were computed from the position data. First, the variability of the displacement of each segment (head, C7, L3) was quantified by computing the standard deviation of the position along the anteroposterior (*SD*_AP_), mediolateral (*SD*_ML_), and vertical (*SD*_V_) axes. These measurements were used to quantify the degree of stability of the head, the cervical segment (upper trunk) and the lumbar segment (lower trunk) in space (the lower the standard deviation of position, the more stable the segment is in space).

**Figure 2:**
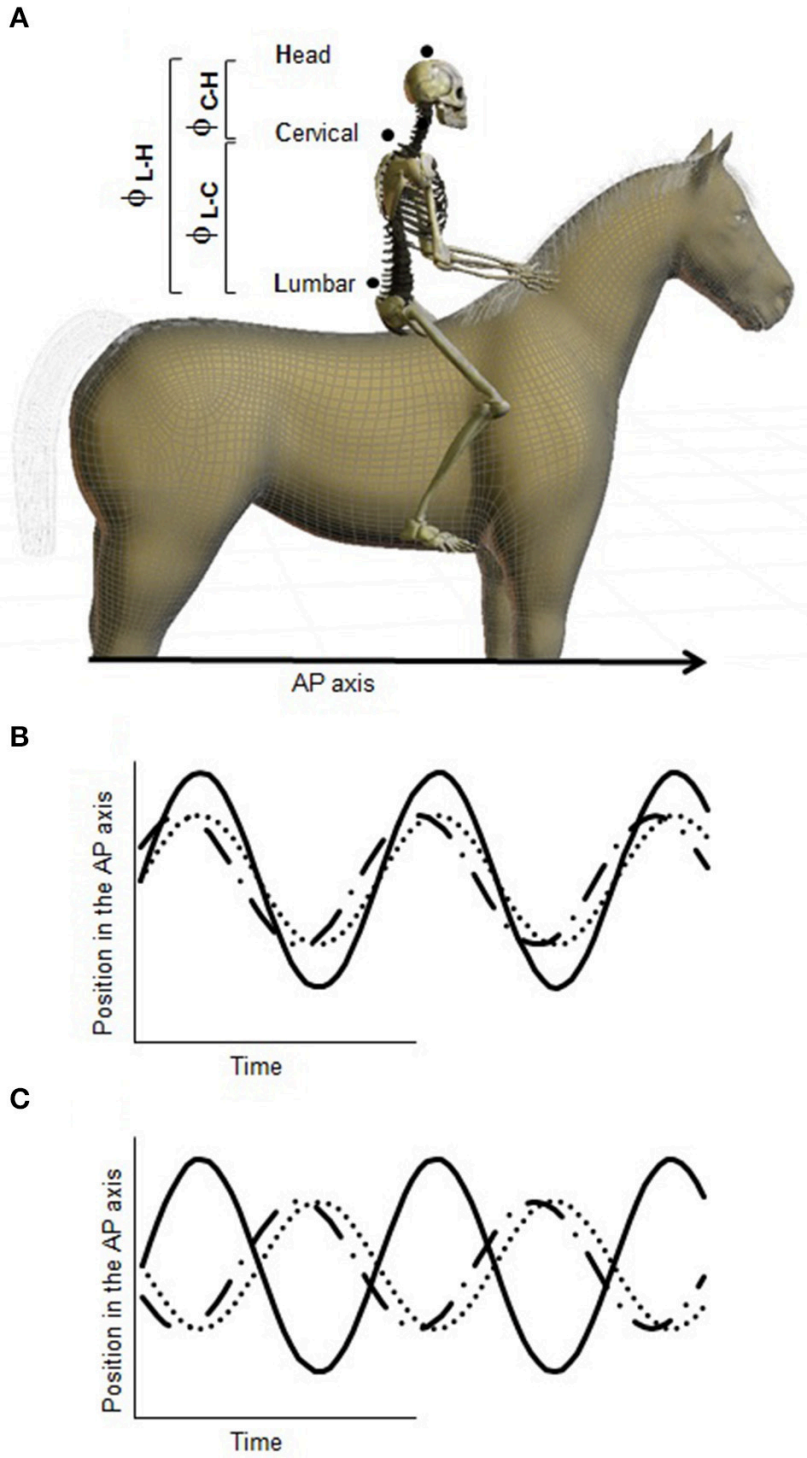
**Illustration of the coordination modes with different relative phase values. (A)** Illustration of the relative phases computed (ϕ_L–C_, ϕ_L–H_, ϕ_C–H_). **(B)** In-phase coordination between the referent segment (bold line) and the segment represented by the dotted line (the segments move synchronously in the same direction resulting in a relative phase of 0°); relative phase of −30° (= 330°) between the referent segment (bold line) and the segment represented by the dash-dotted line. **(C)** Anti-phase coordination (the segments move synchronously in opposite directions, resulting in a relative phase of 180°) between the referent segment (bold line) and the segment represented by the dotted line; relative phase of 150° between the referent segment (bold line) and the segment represented by the dash-dotted line.

Second, we evaluated the coordination modes the riders used to stabilize their posture. Because the control of the hips, trunk and neck impacts mainly the displacements of the upper body in the AP axis, we analyzed postural coordination along this axis. Coordination modes were computed from position data in the AP axis using the mean relative phases ϕ_L–C_, ϕ_L–H_, and ϕ_C–H_ between the lumbar and the cervical segments, the lumbar segment and the head, and the cervical segment and the head, respectively (Figure 2A). The standard deviations of the mean relative phases (*SD*ϕ_L–C_, *SD*ϕ_L–H_, *SD*ϕ_C–H_) were computed as measurements of the within-participants dispersion around the mean relative phase.

Prior to relative phase computation, position data were filtered with a recursive second-order Butterworth filter with a cutoff frequency of 2 Hz. For each pair of segments, we computed the point estimate of relative phase (e.g., Zanone and Kelso, 1992) using the maximum position of each segment in the AP axis for every movement cycle:

(1) $$\begin{array}{l} \phi_{rel}=\frac{\left(t_{1}-t_{2}\right)}{\left(t_{1}-t_{0}\right)}\times 360{}^{\circ} \end{array}$$

where *t*_0_ and *t*_1_ are the time of occurrence of two successive maximum positions along the AP axis for the reference segment, and *t*_2_ is the time of occurrence of maximum position of the second segment. In other words, the time difference between the position peaks of the two segments is expressed in degrees relative to the period of the reference segment.

Each trial (corresponding to one visual condition) comprised about 65 movement cycles. Mean relative phases and their standard deviations were computed in a circular fashion (Batschelet, 1981) over the 65 resulting discrete relative phase values. Figures 2B,C illustrates different relative phase values in the temporal domain. A mean relative phase of 0° (corresponding to an in-phase coordination mode) indicates that the two segments were moving synchronously forward and backward. A relative phase of 180° (corresponding to an anti-phase coordination mode) indicates that the two segments were moving in opposite directions. Other relative phase values indicate a lead or a lag of one segment with respect to the other: for ϕ_L–C_ and ϕ_L–H_, values between 0 and 180° indicate that the movement peak of the lumbar segment preceded the movement peak of the cervical segment and the movement peak of the head, respectively; for ϕ_C–H_, values between 0 and 180° indicate that the movement peak of the cervical segment preceded the movement peak of the head.

Finally, RFT perceptual scores revealing errors in the gravitational vertical estimation due to the tilted frame were computed using Nyborg and Isaksen's method 1974.

### Statistical analysis

Levene's tests were conducted on *SD*_AP_, *SD*_ML_, and *SD*_V_ to assess the homogeneity of variance between the Professional and Club riders for each vision condition (No scene, Cont scene, Strob scene, No vision) and each segment (head, C7, L3). Of the 36 resulting comparisons, only two were significant (at an uncorrected significance level of 0.05) with the standard deviation of the head being higher for the Club riders than for the Professional riders in the AP axis in the No Vision condition (uncorrected *p* = 0.028) and in the ML axis in the Cont scene condition (uncorrected *p* = 0.034). Since the variances of the groups were homogenous overall, we conducted separate Expertise (Professional riders, Club riders) × Segment (Head, Cervical, Lumbar) × Vision (No scene, Cont scene, Strob scene, No vision) ANOVAs with repeated measures on the two last factors on *SD*_AP_, *SD*_ML_, and *SD*_V_. In order to address our second hypothesis, planned comparisons of least-squares means were used to compare specifically head variability between the Professional and Club riders in the different conditions of vision.

Rayleigh Uniformity Tests conducted on ϕ_L–C_, ϕ_L–H_, and ϕ_C–H_ for each trial and each participant revealed that relative phase distributions were significantly directional (i.e., not uniform), *ps* < 0.05. One of the Club riders presented a distribution that did not differ from a uniform distribution in several trials (*ps* > 0.05) and was thus removed from the analyses on coordination modes. Levene's tests conducted on ϕ_L–C_, ϕ_L–H_, and ϕ_C–H_ for each vision condition revealed no significant difference between the variance of the Professional and Club riders (uncorrected *ps* ≥ 0.063). These results, plus the fact that the range of mean relative phase values over participants did not exceed 180° in every experimental condition, allowed us to conduct analyses of variance^2^ on ϕ_L–C_, ϕ_L–H_, ϕ_C–H_, and on their standard deviations. For each variable, we conducted an Expertise (Professional riders, Club riders) × Vision (No scene, Cont scene, Strob scene, No vision) ANOVA with repeated measures on the second factor.

For each significant effect, we conducted *post-hoc* comparisons with corrected *p*-values according to the Holm-Bonferroni procedure. The results were considered significant at the level of 5% and the effect size was estimated using partial eta squared ($\eta_{p}^{2}$).

## Results

### Postural stability

In order to address our main hypotheses with conciseness, we describe the results of the three separate ANOVAs conducted on *SD*_AP_, *SD*_ML_, and *SD*_V_ together for each main effect and each interaction in the following paragraphs (Table 2).

**Table 2:** **Results of the Expertise × Segment × Vision ANOVAs conducted on SD_AP_, SD_ML_, and SD_V_**.

	**SD_AP_**			**SD_ML_**			**SD_V_**		
	***F***	***p***	**$\eta_{p}^{2}$**	***F***	***p***	**$\eta_{p}^{2}$**	***F***	***p***	**$\eta_{p}^{2}$**
Expertise	**5.64**	**0.026**	**0.197**	3.77	0.065	0.141	1.37	0.254	0.056
Segment	2.89	0.066	0.112	**23.56**	**0.000**	**0.506**	**18.68**	**0.000**	**0.448**
Vision	**5.07**	**0.003**	**0.181**	**7.75**	**0.000**	**0.252**	0.76	0.522	0.032
Segment × Expertise	0.14	0.867	0.006	2.76	0.074	0.107	**3.95**	**0.026**	**0.147**
Vision × Expertise	1.76	0.163	0.071	2.00	0.122	0.080	0.91	0.438	0.038
Segment × Vision	**4.86**	**0.000**	**0.175**	**2.73**	**0.015**	**0.106**	0.96	0.453	0.040
Segment × Vision × Expertise	0.46	0.837	0.020	2.16	0.051	0.086	1.21	0.302	0.050

*Significant differences are indicated in bold. $\eta_{p}^{2}$ = Partial Eta Squared*.

The ANOVAs revealed a significant main effect of Expertise on *SD*_AP_ [*F*_(1, 23)_ = 5.64, *p* = 0.026] showing that anteroposterior motion was greater for the Club riders (mean ± SE: 2.58 cm ± 0.09) than for the Professional riders (mean ± SE: 2.27 cm ± 0.10). The main effect of Expertise was not significant for *SD*_ML_ [*F*_(1, 23)_ = 3.76, *p* = 0.065] and *SD*_V_ [*F*_(1, 23)_ = 1.37, *p* = 0.254].

The main effect of Segment did not reach significance for *SD*_AP_ [*F*_(2, 46)_ = 2.89, *p* = 0.066] but was significant for *SD*_ML_ [*F*_(2, 46)_ = 23.56, *p* < 0.001] and *SD*_V_ [*F*_(2, 46)_ = 18.68, *p* < 0.001]. Holm-Bonferroni *post-hoc* tests showed that for both axes, head variability (mean ± SE: *SD*_ML_ = 1.37 cm, ± 0.11; *SD*_Z_ = 5.17 cm, ± 0.09) was greater, *ps* < 0.001, than the variability of the cervical segment (mean ± SE: *SD*_ML_ = 0.86 cm, ± 0.04; *SD*_V_ = 4.76 cm, ± 0.05) and the lumbar segment (mean ± SE: *SD*_ML_ = 0.73 cm, ± 0.04; *SD*_Z_ = 4.71 cm, ± 0.06), which did not differ from each other, *ps* > 0.20.

**Figure 3:**
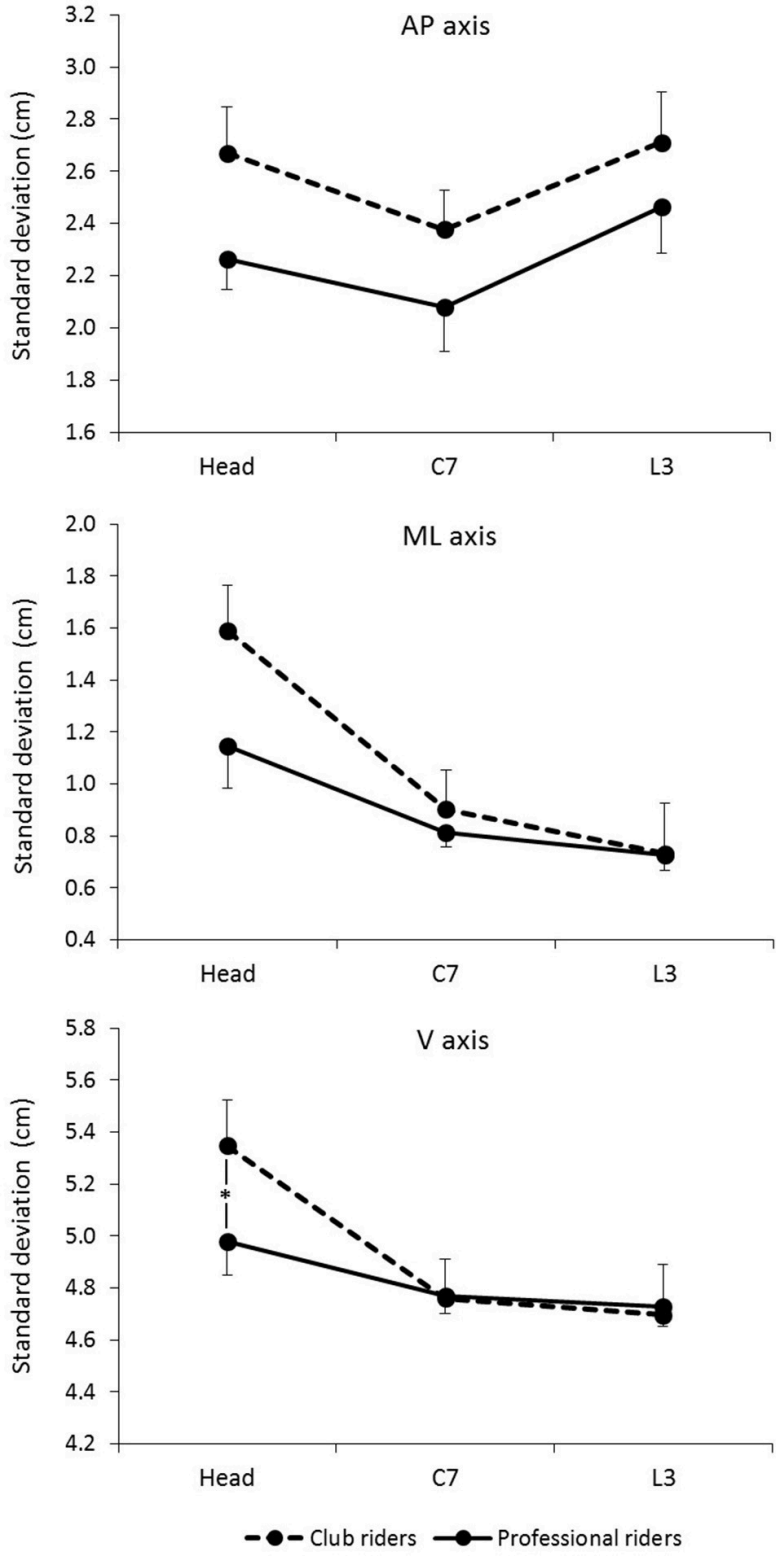
**Mean standard deviation of segment position for the Club riders and the Professional riders in the three axes of movement**. The Expertise × Segment interaction was significant for SD_V_ and not significant for SD_AP_ and SD_ML_. See the text for details. ^*^p < 0.05. The error bars represent standard error.

The Expertise × Segment interaction was significant for *SD*_V_ [*F*_(2, 46)_ = 3.95, *p* = 0.026], revealing that the head was more stable along the vertical axis for the Professional riders (mean ± SE: 4.97 cm ± 0.13) than for the Club riders (mean ± SE: 5.34 cm ± 0.12), *p* = 0.047. No influence of expertise was found for the cervical segment and the lumbar segment, *ps* > 0.95 (Figure 3). The Expertise × Segment interactions were not significant for *SD*_AP_ [*F*_(2, 46)_ = 0.14, *p* = 0.867] and *SD*_ML_ [*F*_(2, 46)_ = 2.76, *p* = 0.074].

The ANOVAs also revealed main effects of Vision on *SD*_AP_ [*F*_(3, 69)_ = 5.07, *p* = 0.003] and *SD*_ML_ [*F*_(3, 69)_ = 7.75, *p* < 0.001], but not on *SD*_V_ [*F*_(3, 69)_ = 0.76, *p* = 0.522]. Holm-Bonferroni *post-hoc* tests showed that *SD*_AP_ was significantly lower in the No scene condition than in the No vision condition, *p* = 0.010, and that *SD*_ML_ was significantly greater in the Cont Scene condition than in the No scene and the No vision conditions, *ps* ≤ 0.007.

**Figure 4:**
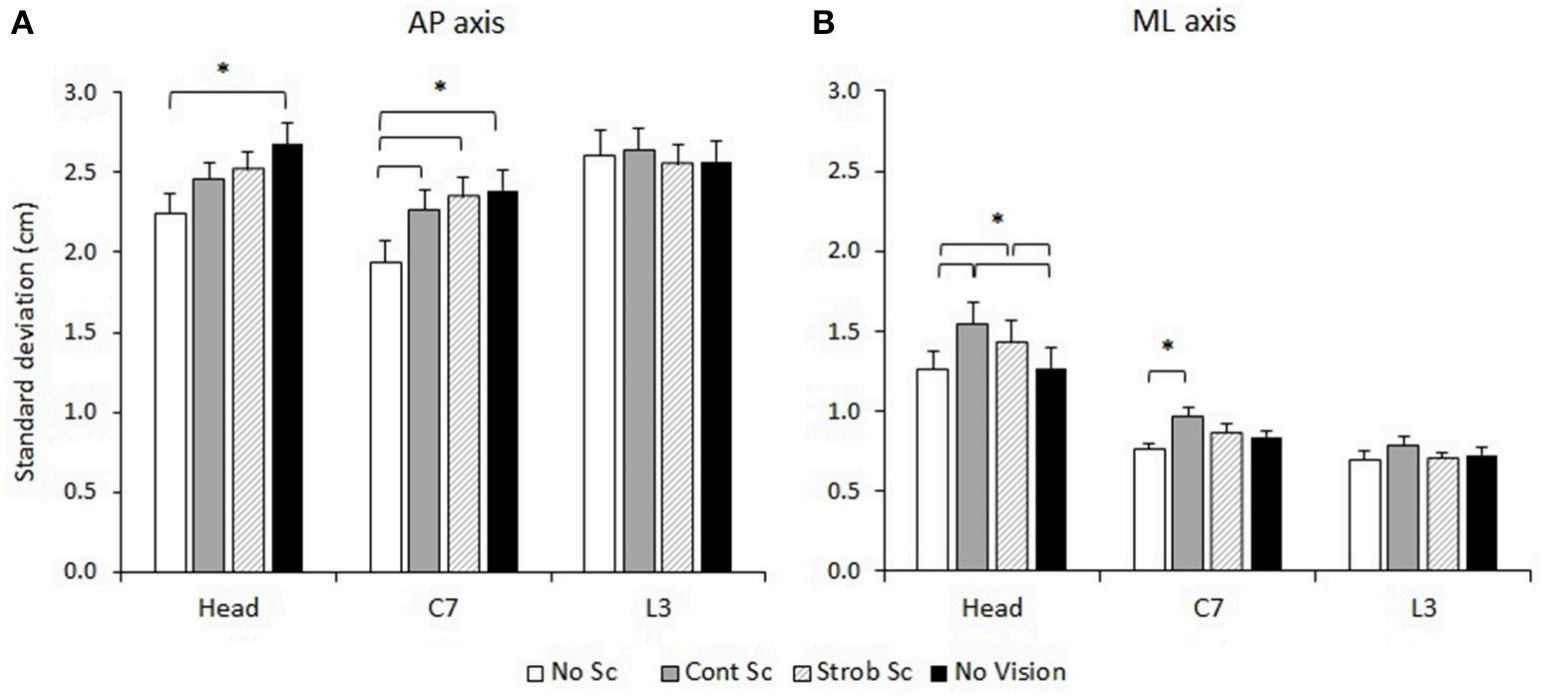
**Segment × Vision interactions on SD_AP_ (A) and SD_ML_ (B)**. No Sc, no simulated scene condition; Cont Sc, continuous simulated scene condition; Strob Sc, stroboscopic simulated scene condition; No vision, No vision condition. ^*^p < 0.05. See the text for details. The error bars represent standard error.

The main effects of Vision can be further specified by the significant Segment × Vision interactions observed on *SD*_AP_ and *SD*_ML_ (Figure 4). For *SD*_AP_, the Segment × Vision interaction [*F*_(6, 138)_ = 4.86, *p* < 0.001] indicated that the vision condition influenced the anteroposterior variability of the head and cervical segment, but had no effect on the lumbar segment (Figure 4A). More precisely, Holm-Bonferroni *post-hoc* tests showed that the No scene condition led to lower anteroposterior variability of head movements than the No vision condition (*p* < 0.001) and that the variability of C7 in the anteroposterior axis was significantly lower in the No scene condition than in all other vision conditions (*ps* ≤ 0.002). There was no other significant difference in segment variability between vision conditions for the AP axis.

For *SD*_ML_, the Segment × Vision interaction [*F*_(6, 138)_ = 2.73, *p* = 0.015] also indicated that lumbar segment variability was not influenced by the vision condition unlike variability of the head and cervical segment (Figure 4B). Holm-Bonferroni *post-hoc* tests conducted on *SD*_ML_ showed that both the Cont scene condition and the Strob scene condition led to greater mediolateral variability of head movements than the No vision and No scene conditions (*ps* ≤ 0.012). Mediolateral variability of the cervical segment was significantly greater in the Cont scene condition than in the No scene condition (*p* = 0.001). There was no other significant difference in segment variability between vision conditions for the ML axis.

Finally, the ANOVA conducted on *SD*_ML_ indicated that the Expertise × Segment × Vision interaction was close to significance [*F*_(6, 138)_ = 2.16, *p* = 0.051]. The Expertise × Segment × Vision interactions were not significant for *SD*_AP_ and *SD*_*V*_ [*Fs*_(6, 138)_ ≤ 1.21, *ps* ≥ 0.302], and there were no significant Vision × Expertise interactions in any axis of movement [*Fs*_(3, 69)_ ≤ 2.00, *ps* ≥ 0.122].

### Contribution of visual information to head stability depending on expertise

#### Effect of expertise on head stability

**Figure 5:**
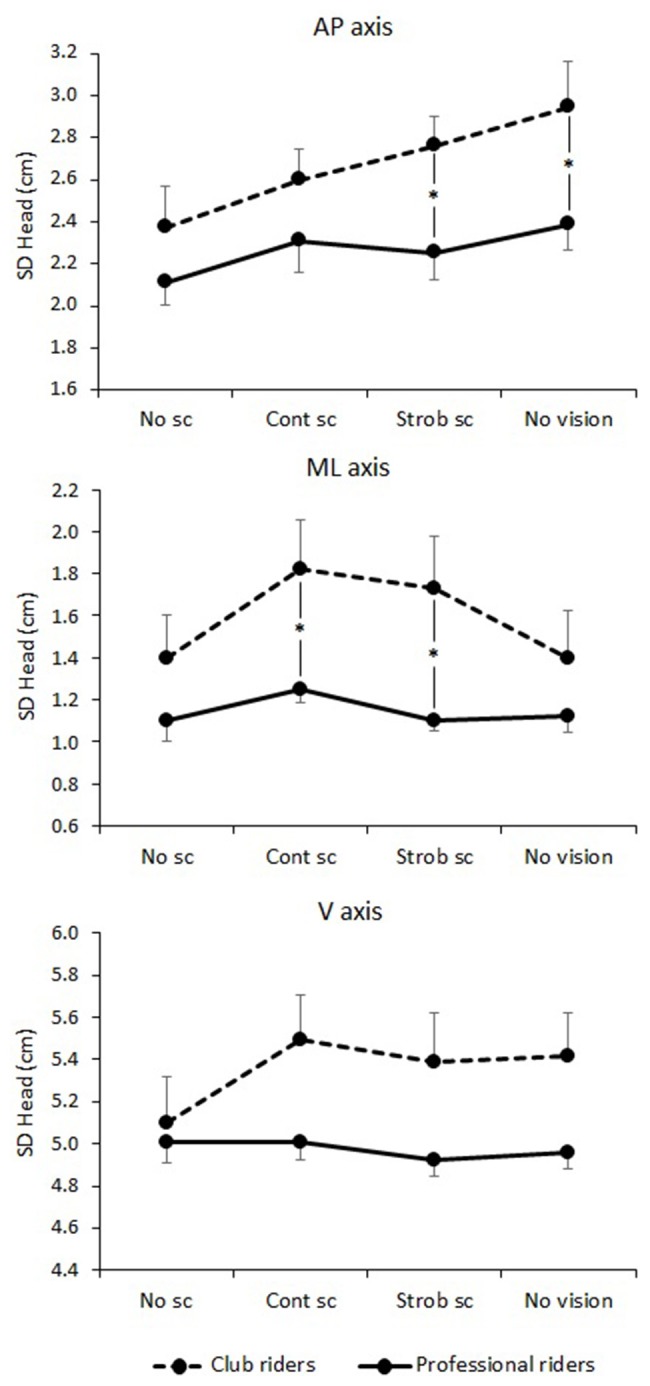
**Head variability depending on vision condition and expertise for the three axes of movement**. No sc, no simulated scene condition; Cont sc, continuous simulated scene condition; Strob sc, stroboscopic simulated scene condition; No vision, No vision condition. ^*^p < 0.05. See the text for details. The error bars represent standard error.

The ANOVAs presented in the previous section revealed a main effect of Expertise on *SD*_AP_ and some interactions involving Expertise for *SD*_ML_ and *SD*_V_. In order to specifically address our second hypothesis that head variability would be lower for the Professional riders compared to the Club riders under visually altered conditions, we conducted planned comparisons of least-squares means on *SD*_AP_, *SD*_ML_, and *SD*_V_ for the head only for each vision condition (Figure 5).

We found that the position of the head was significantly less variable for the Professional riders than for the Club riders in the anteroposterior axis (*SD*_AP_) in the Strob scene [*F*_(23)_ = 7.37, *p* = 0.012] and in the No Vision [*F*_(23)_ = 4.93, *p* = 0.036] conditions. In the mediolateral axis, the position of the head was significantly less variable for the Professional riders than for the Club riders in the Cont scene [*F*_(23)_ = 5.09, *p* = 0.033] and in the Strob scene [*F*_(23)_ = 5.60, *p* = 0.026] conditions. In the vertical axis, the difference between Professional and Club riders was close to significance in the Strob scene condition [*F*_(23)_ = 3.93, *p* = 0.059], in the No Vision condition [*F*_(23)_ = 3.62, *p* = 0.070] and in the Cont scene condition [*F*_(23)_ = 3.57, *p* = 0.072]. Other differences were not significant, *ps* ≥ 0.186.

#### Visual field dependence-independence and head stability

A *t*-test comparing the RFT perceptual scores of the Professional and Club riders revealed a significant difference between the two groups [*t*_(23)_ = 4.53, *p* = 0.044]. The 13 Club riders achieved a mean error of 7.30° (SE ± 1.35°) and the 12 Professional riders a mean error of 4.13° (SE ± 0.49°). These results indicated that the Professional riders were less dependent on the visual field than were the Club riders.

To evaluate the relation between perceptual style and postural stability, we performed Pearson's correlation analyses between RFT scores and head variability. We found significant positive correlations between the standard deviations of the head in the ML axis and the RFT scores in all conditions of vision: Cont scene [*r*_(24)_ = 0.54, *p* < 0.001], Strob scene [*r*_(24)_ = 0.55, *p* < 0.001], No scene [*r*_(24)_ = 0.39, *p* < 0.001], and No vision [*r*_(24)_ = 0.23, *p* = 0.014]. We also found a significant positive correlation between head stability along the V axis and RFT scores in the Cont scene [*r*_(24)_ = 0.15, *p* = 0.048] and No vision [*r*_(24)_ = 0.23, *p* = 0.013] conditions. Finally, no significant correlation was found between head displacements along the AP axis and RFT scores.

### Coordination modes

**Figure 6:**
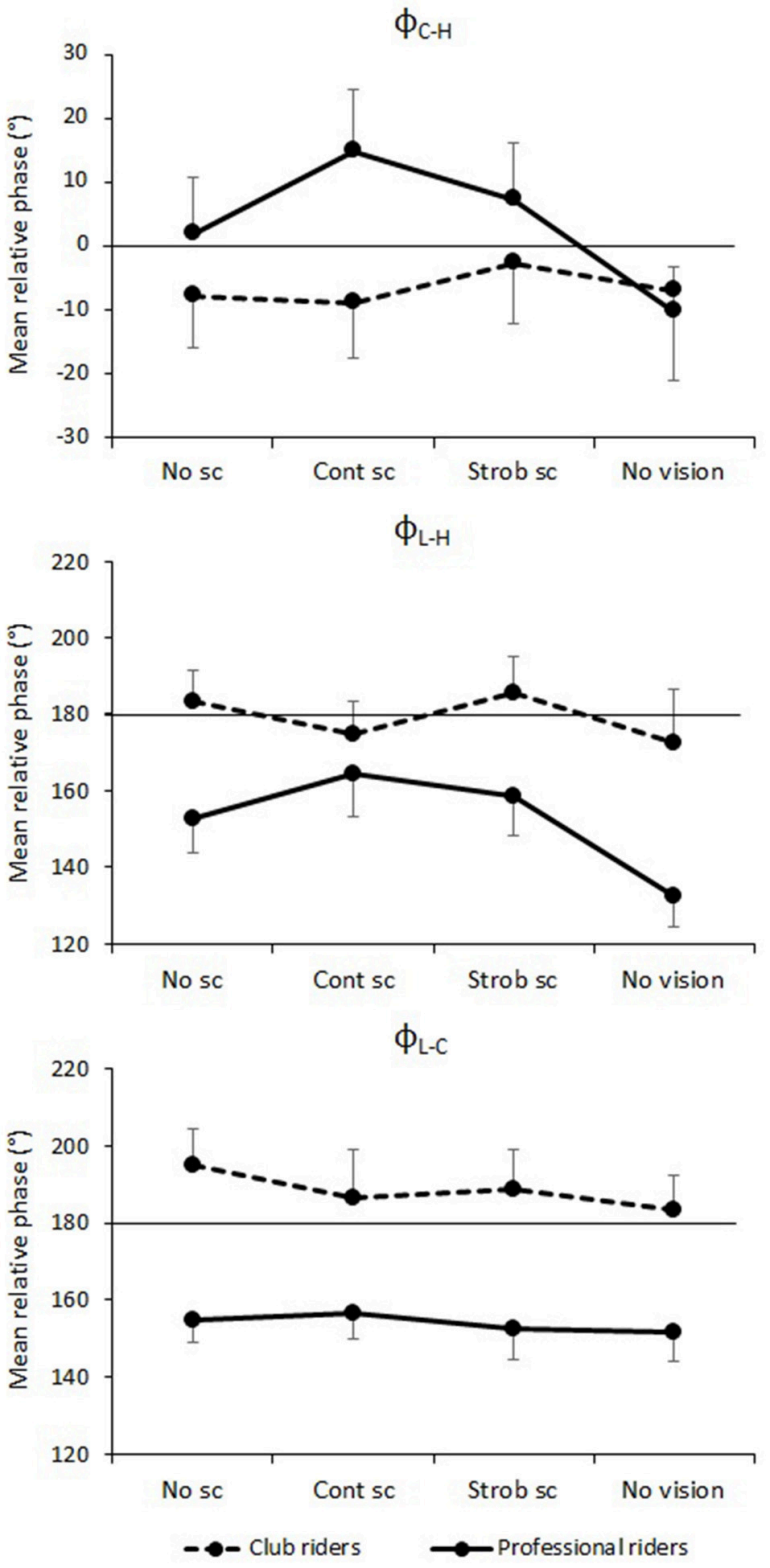
**Mean relative phases in degrees between the cervical segment and the head (ϕ_C–H_), between the lumbar segment and the head (ϕ_L–H_), and between the lumbar and the cervical segments (ϕ_L–C_) according to visual condition and expertise**. No sc, no simulated scene condition; Cont sc, continuous simulated scene condition; Strob sc, stroboscopic simulated scene condition; No vision, No vision condition. See the text for statistical significance. The error bars represent standard error.

Analyses of the variability of segment position revealed significant differences between the Professional and Club riders. In particular, the Professional riders were found to stabilize their head better in the anteroposterior axis than the Club riders. The following analyses were conducted to assess whether this better stabilization was associated with specific postural coordination modes in the anteroposterior axis. For each variable, we conducted an Expertise × Vision ANOVA with repeated measures on the second factor. The results of these analyses are reported in Table 3. Figure 6 presents the mean relative phases for the Club and Professional riders in the four vision conditions.

**Table 3:** **Results of the Expertise × Vision ANOVAs conducted on the mean relative phases and their standard deviations**.

	**Lumbar-Cervical (ϕ_L–C_)**			**Lumbar-Head (ϕ_L–H_)**			**Cervical-Head (ϕ_C–H_)**		
**Mean (ϕ)**	***F***	***p***	**$\eta_{p}^{2}$**	***F***	***p***	**$\eta_{p}^{2}$**	***F***	***p***	**$\eta_{p}^{2}$**
Expertise	**8.20**	**0.009**	**0.271**	2.66	0.117	0.108	0.66	0.427	0.029
Vision	1.77	0.161	0.074	**4.59**	**0.006**	**0.173**	**3.09**	**0.033**	**0.123**
Expertise x Vision	0.99	0.401	0.043	2.24	0.092	0.092	**3.18**	**0.030**	**0.126**
**Standard deviation (*SD*ϕ)**									
Expertise	0.15	0.694	0.007	0.08	0.775	0.004	0.42	0.522	0.019
Vision	**3.26**	**0.027**	**0.129**	1.33	0.271	0.057	2.53	0.064	0.103
Expertise x Vision	0.19	0.898	0.009	**4.95**	**0.004**	**0.184**	**7.08**	**0.000**	**0.243**

*Significant differences are indicated in bold. $\eta_{p}^{2}$ = Partial Eta Squared*.

#### Coordination between the lumbar and cervical segments (ϕ_L–C_ and *SD*ϕ_L–C_)

The ANOVA conducted on ϕ_L–C_ revealed a main effect of Expertise [*F*_(1, 22)_ = 8.20, *p* = 0.009] with a mean relative phase of 188.27° (*SD*ϕ_L–C_ = 24.24°) for the Club riders and of 153.99° (*SD*ϕ_L–C_ = 22.67°) for the Professional riders. This result shows that unlike the Club riders who exhibited an anti-phase pattern between the lower and the upper trunk, the movement cycle of the upper trunk appeared sooner after the movement cycle of the lower trunk in the Professional riders. The main effect of Vision [*F*_(3, 66)_ = 1.77, *p* = 0.161] and the Expertise × Vision interaction [*F*_(3, 66)_ < 0.99, *p* = 0.043] were not significant.

The ANOVA conducted on *SD*ϕ_L–C_ revealed a main effect of Vision on the within-participants dispersion around the mean relative phase [*F*_(1, 66)_ = 3.26, *p* = 0.027]. However, Holm-Bonferroni *post-hoc* tests revealed no significant difference between vision conditions from pairwise comparisons, *p* ≥ 0.18. The main effect of Expertise [*F*_(1, 22)_ < 0.15, *p* = 0.694] and the Expertise × Vision interaction were not significant [*F*_(3, 66)_ < 0.19, *p* = 0.898].

#### Coordination between the lumbar segment and the head (ϕ_L–H_ and *SD*ϕ_L–H_)

The ANOVA conducted on ϕ_L–H_ revealed a main effect of Vision [*F*_(3, 66)_ = 4.59, *p* = 0.006]. Holm-Bonferroni *post-hoc* tests showed a significant difference between the coordination modes in the No vision condition (ϕ_L–H_ = 152.4 ± 47.16°) and the Strob scene and No scene conditions, *ps* ≤ 0.050, with mean relative phases of 172.16° (± 42.59°) and 168.01° (± 44.36°) for the Strob scene and the No scene conditions, respectively. No other difference between vision conditions was significant. The main effect of Expertise [*F*_(1, 22)_ = 2.66, *p* = 0.117] and the Expertise × Vision interaction were not significant [*F*_(3, 66)_ = 2.24, *p* = 0.092].

The ANOVA conducted on *SD*ϕ_L–H_ showed no main effect of Expertise [*F*_(1, 22)_ < 1, *p* = 0.77] and Vision [*F*_(3, 66)_ = 1.33, *p* = 0.27], but revealed a significant Expertise × Vision interaction [*F*_(3, 66)_ = 4.95, *p* = 0.004]. However, Holm-Bonferroni *post-hoc* tests revealed no significant difference from pairwise comparisons (*ps* ≥ 0.156).

#### Coordination between the cervical segment and the head (ϕ_C–H_ and *SD*ϕ_C–H_)

The ANOVA conducted on ϕ_C–H_ revealed a main effect of Vision [*F*_(3, 66)_ = 3.09, *p* = 0.033] and a significant Vision × Expertise interaction [*F*_(3, 66)_ = 3.18, *p* = 0.030]. Holm-Bonferroni *post-hoc* tests showed that ϕ_C–H_ differed between the No vision (mean ϕ_C–H_ = −10.30°, *SD* ± 24.66°) and the Cont scene (mean ϕ_C–H_ = 14.74°, *SD* ± 34.13°) conditions for the Professional riders only (*p* = 0.037), and that no other difference between vision conditions was significant for either group of participants. The main effect of Expertise was not significant [*F*_(1, 22)_ < 0.66, *p* = 0.427].

The ANOVA conducted on *SD*ϕ_C–H_ showed no main effect of Expertise [*F*_(1, 22)_ < 1, *p* = 0.52] or Vision [*F*_(3, 66)_ = 2.53, *p* = 0.064]. However, the Vision × Expertise interaction was significant [*F*_(3, 66)_ = 7.08, *p* < 0.001]. Holm-Bonferroni *post-hoc* tests specified that the within-participants dispersion around the mean relative phase was lower in the No vision condition than in the Cont scene condition (*p* = 0.033) and in the No scene condition (*p* = 0.036) for the Professional riders only.

## Discussion

The purpose of this study was to evaluate the relative contribution of visual information to head and trunk stability in Club and Professional horseback riders and the coordination modes adopted to regulate balance according to expertise. Modes of spatial referencing were taken into account to determine the riders' perceptual typology and its possible relation to postural stability.

Before addressing our main hypotheses, it should be noted that overall expertise levels and vision conditions, the head exhibited larger displacements than the upper trunk and the lower trunk in the mediolateral and vertical axes. We did not find such an effect of body segment along the anteroposterior axis: the displacements of the head, the upper trunk and the lower trunk remained in the same range of motion in the AP axis. This suggests that the inverted pendulum model of balance often used to describe quiet standing (e.g., Winter et al., 1997; Peterka, 2002) does not apply to postural regulation in horseback riding. This is a first indication that more complex coordination patterns take place to maintain balance in response to the perturbations induced by the simulator's movements.

We can also note several general results independent of expertise regarding the influence of vision condition on the stability of the different levels of the spine. First, we observed no influence of the vision condition on the variability of the lumbar segment. This result suggests, unsurprisingly, that the stability of the lower trunk is influenced mainly by the mechanical perturbations of the support surface and not by the visual information available. Second, the head and the cervical segments were significantly more stable in the AP axis when the riders faced the white projection screen (No scene condition) than in the other vision conditions. This result is in accordance with previous research that showed the stabilizing effect of fixed surroundings (e.g., Lee and Lishman, 1975; Guerraz et al., 2001). Third, we found no influence of the vision condition on postural stability along the V axis. A likely explanation is that the magnitude of the mechanical perturbations induced by the movements of the riding simulator along the vertical axis has much more influence on postural stability than the visual information available.

### Postural stability and horseback riding expertise

Our first hypothesis was that Professional riders would produce lower postural displacements and deploy more efficient postural control from the top of the head to the lower trunk, leading them to better stabilize their head. Our findings support this hypothesis. First, riding expertise appears to be characterized by the ability to minimize postural displacements in the anteroposterior axis. Overall body segments (i.e., head, upper trunk, and lower trunk) and vision conditions, the Professional riders exhibited greater postural stability in the anteroposterior axis than the Club riders. Overall vision conditions, the Professional riders also exhibited greater head stability in the V axis than the Club riders, while there was no significant difference in the stability of the upper trunk and the lower trunk based on the level of expertise. These findings suggest that postural stability of the overall upper body in the AP axis and more specifically of the head in the V axis is a signature of expertise in horseback riding. However, a closer look at the results according to vision conditions is needed to specify the influence of expertise on postural stability.

### Relative contribution of sensory information to riders' postural stability depending on expertise

Our second hypothesis was that the contribution of visual information to riders' postural stability is reduced among expert riders in favor of vestibular and somesthetic reliance, leading the experts to maintain head stability better in visually altered conditions. Based on our hypothesis, we expected the selective or total suppression of static and dynamic visual cues to induce greater displacements of the head in the Club riders than in the Professionals.

We found no difference in head stability between the Professional and the Club riders in the two unrestricted visual conditions (Cont scene and No scene) for the different axes of movement (AP, ML, V). The only exception was head variability in the ML axis in the Cont scene condition, with the Club riders exhibiting greater head displacements than the Professional riders. One possible explanation for this result might be that Club and Professional riders use different strategies during the turns simulated by the visual scene. Indeed, the riding simulator reproduces the perturbation of the chosen pace (gallop in our case) with a fixed orientation of the mechanical horse. Thus, unlike a real ride, the back of the mechanical horse does not turn in the horizontal plane when the visual scene indicates a curved trajectory of the route. The riders might have dealt with this discrepancy differently depending on their level of expertise. It is also possible that the Club riders did not use the visual information provided by the simulated scene with sufficient efficiency to stabilize their head in the ML axis as well as the Professional riders. Solving this question would necessitate a coupling between movement measures and data from the simulated visual scene that is not currently available with the Persival simulator. This coupling would represent a major evolution of the apparatus for research purposes.

The results obtained under stroboscopic illumination were in accordance with our hypothesis. In this condition, the Club riders exhibited greater head displacements compared to the Professionals. The difference was significant in the AP and ML axes, and close to significance in the vertical axis. Dynamic visual cues have been shown to play a major role in postural stabilization during upright stance (e.g., Amblard and Crémieux, 1976; Amblard and Carblanc, 1980; Amblard et al., 1985). In particular, postural stability was found to be severely impaired under stroboscopic illumination (Amblard and Crémieux, 1976; Paulus et al., 1984; Amblard et al., 1985; Assländer et al., 2015), though with some interindividual differences (Crémieux and Mesure, 1994; Isableu et al., 2010, 2011). The present findings highlight these interindividual differences in relation to sports expertise. Suppressing dynamic visual cues differently affected head stability depending on the level of expertise in horseback riding: the Club riders appear to rely more on dynamic visual cues than the Professionals in order to control head stability.

The results obtained in the absence of visual information also agreed with our hypothesis. The position of the head along the AP axis was less variable for the Professional riders than for the Club riders in the No vision condition. However, we found no significant difference along the ML and V axes. These findings reveal that experts handle the absence of visual information better than less experienced riders to maintain head stability in the AP axis.

In order to complete our analysis on the relative contribution of sensory information to postural stability, we examined the relations between perceptual typologies, postural stability and expertise in horseback riding. The perceptual scores obtained with the RFT revealed that the Professional riders were less dependent on the visual field than the Club riders. This predominant perceptual typology in experts agrees with previous findings in other physical activities (e.g., Golomer et al., 1999; Liu, 2003; Guillot and Collet, 2004; Rousseu and Crémieux, 2005) and, in line with our second hypothesis, suggests that expert horseback riders rely more on vestibular and somesthetic information than less experienced riders. This enhanced reliance on non-visual information likely explains the greater head stabilization observed in the Professional riders in visually altered conditions (i.e., No vision and Strob scene conditions) compared to the Club riders (Isableu et al., 2003, 2010; Brady et al., 2012). Correlation analyses showed that field dependence–independence scores measured with the RFT were positively correlated with the variability of the head along the ML and V axes. This result provides an additional demonstration of the effect of perceptual typology on postural control (e.g., Isableu et al., 2010, 2011).

The joint analysis of postural stability and perceptual typology highlights a major result of the present study. Except for the mediolateral variability of the head in the Cont scene condition discussed above, the Club riders managed to maintain a level of head stability similar to the Professional riders when visual information was available, whether the visual environment was fixed (No scene condition) or simulated displacements corresponding to a ride (Cont scene condition). In fact, in these unrestricted visual conditions, the Club riders could rely on visual information to stabilize their head in space. Given that the Club riders tended to be field-dependent, the postural task was considerably harder in visually altered conditions. The Club riders appeared unable to reweight the sensory information to respond to the lack of visual cues. In contrast, the Professional riders, who were more field-independent, were able to maintain a high level of head stability regardless of the availability of visual information. These results strongly suggest that the capacity to reweight the relative contribution of different sensory information depending on environmental conditions is a more prominent indicator of expertise in horseback riding than the rider's postural stability in unrestricted visual conditions. However, this conclusion does not tell us if there are different sensorimotor processes involved in expert and non-expert riders to reach the same level of head stability in unrestricted conditions of vision. The experts' ability to use vestibular and somesthetic information in altered visual conditions does not necessarily mean that they do not use visual information when it is available. Also, the fact that head stability was equivalent in experts and non-experts does not necessarily mean that they adopted similar motor behaviors to achieve this level of stability. Fully addressing these questions would necessitate a complete specific design (e.g., Oie et al., 2001; Peterka, 2002). Nevertheless, the present results on postural coordination modes do provide initial insights.

### Postural coordination modes

Our third hypothesis was that horseback riding experts would exhibit specific coordination modes to maintain a high level of postural stability. Research has shown that when two joints oscillate together, they are strongly attracted toward in-phase (relative phase close to 0°) or anti-phase (relative phase close to 180°) patterns. These two attractive states have been identified in numerous joint pairings, including bimanual (e.g., Yamanishi et al., 1980; Kelso, 1984), arm-leg (e.g., Kelso and Jeka, 1992), elbow-wrist (e.g., Kelso et al., 1991), ankle-wrist (e.g., Carson et al., 1995), and ankle-hip (e.g., Bardy et al., 1999; Faugloire et al., 2005). The execution of patterns differing from in-phase and anti-phase often requires intensive practice (e.g., Zanone and Kelso, 1992; Faugloire et al., 2006, 2009). The results of the present study demonstrate that these spontaneous modes found in many effector systems are also found in the postural coordination of horseback riders in the anteroposterior axis: The Club riders exhibited an anti-phase coordination mode (segments moving synchronously in opposite directions) both between the lower trunk and the upper trunk and between the lower trunk and the head, and an in-phase coordination mode (segments moving synchronously forward and backward) between the upper trunk and the head.

Interestingly, the Professional riders adopted coordination modes that departed from pure in-phase and anti-phase patterns. In particular, they exhibited a mean relative phase of 154° between the lower and the upper trunk (ϕ_L–C_) in every vision condition, while the Club riders adopted an anti-phase pattern (mean ϕ_L–C_ = 188°). In other words, the Professional riders anticipated the anteroposterior movements of the upper trunk compared to the Club riders: the maximal position of the cervical segment occurred sooner in the Professional riders than in the Club riders, for whom the maximal position of the cervical segment (forward) was almost perfectly synchronized with the minimal position of the lumbar segment (backward).

Another interesting result is that, unlike the Club riders, the Professional riders exhibited changes in coordination modes between the cervical segment and the head depending on available visual information. The Club riders exhibited a close to in-phase coordination mode with a slight lag of the cervical segment with respect to the movement of the head (indicated by the negative values of the relative phases) in every vision condition (mean ϕ_C–H_ ranging from −8.83° in the Cont scene condition to −2.75° in the Strob scene condition). By contrast, the Professional riders adopted coordination patterns ranging from +14.74° in the Cont scene condition to −10.30° in the No vision condition (Figure 6). When the simulated scene was projected on the screen, the Professional riders anticipated the cervical segment's movements with respect to the head's movements (positive ϕ_C–H_ of +14.74° in the Cont scene condition). When no visual information was available, the Professional riders adopted a coordination pattern similar to the Club riders with the movement of the cervical segment following the head movement with a slight delay (negative ϕ_C–H_ of −10.30° in the No vision condition). Again, it is interesting to note that this cervical-head coordination mode observed when no visual information was available was also adopted by the Club riders in every other vision condition. This result reveals that, although the Professional riders were able to stabilize their head better than the Club riders in visually altered conditions, they did not rely only on vestibular and somesthetic information in unrestricted visual conditions. The specific relative phase between the cervical segment and the head exhibited by the Professional riders in the Cont scene conditions suggests that the experts did use visual information in this condition to adapt their postural coordination modes to environmental conditions. This interesting result is difficult to discuss further and would have to be connected to other dependent variables such as the flexion-extension of the neck and the visual search behavior of the participants.

Finally, we found that the coordination between the lumbar segment and the head (ϕ_L–H_) depended on the vision condition over the two levels of expertise, with a significant difference between the No vision condition (ϕ_L–H_ = 152.42°) and every other vision condition (ϕ_L–H_ ranging from 168.01° in the No scene condition to 172.16° in the Strob scene condition). Figure 6 (middle panel) strongly suggests that this effect of vision is due mainly to a change in the coordination mode of the Professional riders who exhibited relative phases between the lumbar segment and the head ranging from 164.33° in the Cont scene condition to 132.42° in the No vision condition, while the Club riders maintained an anti-phase coordination mode in every vision condition. This result is likely to be the direct consequence of the behavior observed on the two underlying levels of lumbar-head coordination, namely lumbar-cervical and cervical-head coordination.

### Limitations and perspectives

The present study is the first to investigate the contribution of visual information to postural stability and the postural coordination modes in horseback riding depending on expertise. As such, several experimental features can potentially be enhanced in future work. Using an equestrian simulator enabled us to overcome difficulties encountered in studies on horseback riding such as controlling and reproducing the horses' movements, facilitating data collection, and manipulating vision conditions. However, while the projected visual scene simulated the horizontal displacements corresponding to a ride, including turns, the mechanical horse's orientation was fixed. Therefore, while the riding simulator faithfully reproduced the vertical and pitch perturbations of the chosen pace, it did not produce any horizontal movements, either in yaw or in translation. Consequently, the mechanical perturbations were only partially similar to a real ride. In addition, there was a discrepancy between the visual information provided by the projected visual scene and the mechanical horse's motion. Given the current technical constraints of riding simulators, it seems that only a study in the field could overcome these limitations, though with all the other difficulties that this method would entail. A related issue concerns the possibility to match precisely the data from the projected visual scene and movement measures. This coupling would help understand more finely how riders react to the different events simulated in the visual scene, such as the aforementioned turns. This might have helped interpret the difference in head stability in the mediolateral axis between the Professional and Club riders that we observed only when the simulated visual scene was projected.

Another limitation concerns the size of the visual field occupied by the projected scene. The simulated scene used in the present study was limited to the participants' central visual field, thereby reducing the possibility of using peripheral vision which is known to play an important role in postural control (e.g., Pavard et al., 1976; Amblard et al., 1985). A multi-sided immersive environment would make it possible to strengthen and extend the conclusions of the present study. Another, non-exclusive, perspective would be to collect and combine additional data about pelvis orientation, angular displacements (of the hips, neck and knees), and/or horse-rider interactions, for example. Indeed, the new insights we have gained through the study of horseback riders' postural control encourage further investigations to better understand the highly complex task that is horseback riding.

## Conclusions

The present study demonstrates a differential contribution of visual information to postural stability in horseback riding depending on expertise. First, compared to the Club riders, the Professional riders exhibited greater head stability in the anteroposterior axis when vision conditions were altered. Second, RFT perceptual scores revealed that the Professional riders were less dependent on the visual field than the Club riders. Third, we found that the more dependent the riders were on the visual field, the greater their head variability. These results suggest that expert horseback riders rely more on vestibular and somesthetic information to stabilize their head in space than less experienced riders. Our assessment of the coordination modes between the different levels of the spine completes and specifies this conclusion. Unlike the Club riders, who exhibited similar in-phase or anti-phase patterns in the different vision conditions, the Professional riders exhibited changes in coordination modes depending on the visual information available. Thus, even though the expert riders proved to be less dependent on visual information to stabilize their head than the non-expert riders, they appeared to make use of visual information when it was available to adapt their postural coordination modes. The combination of stability, perceptual typology and postural coordination measures therefore strongly suggests that expert riders are better able to reweight sensory information in order to control their posture according to task constraints.

## Author contributions

AO, LL, and EF designed the study. AO and SB performed the experiments. AO and EF analyzed the data and AO, EF, LL, and BI wrote the manuscript.

## Funding

Part of data analysis and the writing of this paper was supported by the “Institut Français du Cheval et de l'Equitation” (IFCE) and the “Fonds Eperon” as part of the “RiderFeel” proposals.

### Conflict of interest statement

The authors declare that the research was conducted in the absence of any commercial or financial relationships that could be construed as a potential conflict of interest.

## Footnotes

^1^In the dynamical systems perspective, stability has a precise definition related to a system's response to a change in initial conditions or to a perturbation (e.g., Strogatz, 1994). In this view, the term postural stability refers to the stability of the underlying movement dynamics (e.g., Newell et al., 1993). In the present paper, we use the term “postural stability” in its most widespread sense in the literature on postural control, which supposes a reduced amount of variability of the segments in space.

^2^When the range of distribution does not exceed 180°, circular statistics (Batschelet, 1981; Mardia and Jupp, 2000) and regular linear statistics result in virtually identical outcomes.
